# The prevalence of HBV infection in the cohort of IDPs of war against terrorism in Malakand Division of Northern Pakistan

**DOI:** 10.1186/1471-2334-11-176

**Published:** 2011-06-20

**Authors:** Fawad Khan, Haji Akbar, Muhammad Idrees, Hayat Khan, Khuram Shahzad, Mahmood A Kayani

**Affiliations:** 1 Department of Biotechnology, University of Malakand, Pakistan; 2 National Centre of Excellence in Molecular Biology, Lahore, Pakistan; 3 Department of Biosciences, COMSATS Institute of Information Technology Islamabad Campus, Pakistan; 4 Department of Animal Sciences, University of Illinoise Urbana Champaign, USA

**Keywords:** HBV, Gender disparity, Risk factors, Prevalence, Malakand Division

## Abstract

**Background:**

Hepatitis B is an important public health problem in the Pakistani population and is the major cause of chronic hepatitis, cirrhosis, fibrosis and hepatocellular carcinoma. High prevalence of HBV infections has been observed especially in areas of low economic status. In spite of effective immunization programs, no significant change has been observed in the epidemiology of HBV in the rural areas of Pakistan (~67.5% of the total population) mainly due to lack of interest from government authorities and poor hygienic measures. The current study was aimed at estimating the prevalence and risk factors associated with HBV infection within internally displaced persons (IDPs) due to war against terrorism in the Malakand Division of Northern Pakistan.

**Methods:**

Blood samples from 950 IDPs suspected with HBV infection (including both males and females) were collected and processed with commercial ELISA kits for HBsAg, Anti HBs, HBeAg, Anti HBe antibodies. The samples positive by ELISA were confirmed for HBV DNA by real-time PCR analysis.

**Results:**

The overall prevalence of HBV observed was 21.05% of which 78.5% were males and 21.5% were females. Most confirmed HBV patients belong to the Malakand and Dir (lower) district. High-risk of infection was found in the older subjects 29.13% (46-60 years), while a lower incidence (11.97%) was observed in children aged <15 years. Lack of awareness, socioecomic conditions, sexual activities and sharing of razor blades, syringes and tattooing needles were the most common risk factors of HBV infection observed during the cohort of patients.

**Conclusion:**

The present study, revealed for the first time a high degree of prevalence of HBV infection in rural areas of Northern Pakistan. The noticed prevalence is gender- and age-dependent that might be due to their high exposures to the common risk factors. To avoid the transmission of HBV infection proper awareness about the possible risk factors and extension of immunization to the rural areas are recommended.

## Background

Hepatitis B is an important public health concern in both developing and developed countries affecting approximately 3.5 billion of the world's population and additionally ≥ 400 million are chronic carriers [1-4]. It has been estimated globally that each year ~1-2 million people die from HBV related complications such as chronic hepatitis, cirrhosis hepatocellular carcinoma (HCC) [2-8]. HBV is endemic in the Pakistani population with a rate of 3% HBV carriers in the country. Although the rate of exposure to HBV in Pakistan is not fully confirmed, Awan *et al *(2010) reported ~38% prevalence with a 4% carrier rate and 32% with anti-HBV surface antibodies by natural conversion [3].

The highest concentrations of infectious HBV are in blood, serum and serum-derived body fluids, such as semen and saliva [9]. It has been reported earlier in 2002 that the hepatitis B virus can live for several days in dried blood on table surfaces, needles, syringes and razors [10,11]. HBV transmission has been observed by percutaneous or mucosal exposure to infected blood and body fluids [12]. Transmission also occurs via the use of unsterilized dental and surgical instruments, shaving from barber, reuse of needle for nose and ear piercing, reuse of disposable syringes and sharing needles with drugs addicts, sharing personal things such as razors, toothbrushes, and nail cutters, sexual and prolonged close personal contact with infected personnel [13]. High prevalence of HBV was observed in geographical areas of low economic status, which underscores the importance in controlling this disease because ~67.5% of the Pakistani population belongs to rural areas of low economic status [14,15].

This study was planned to evaluate the presence of HBV in internally displaced persons (IDPs) due to war against terrorism in Malakand Division, a backward rural area in Pakistan. The study also evaluated the potential risk factors predisposing this population to HBV. It is anticipated that this study will help in creating awareness among the people about the potential risk factors in order to avoid the possible transmission of hepatitis B infection.

## Methods

### Description of the Study Area

Pakistan is a federation of four provinces (Punjab, Sindh, Khyber PukhtoonKhwa, and Balochistan), a capital territory and federally administered tribal areas. Malakand Division is an important division of Khyber Pukhtoonkhwa which includes the districts Swat, Buner, Shangla, lower Dir, upper Dir, Chitral and Malakand. In the locality, the majority of the population is composed of Pashtuns (locally referred to as Pakhtuns) and other smaller ethnic groups. The principal language is Pushto (locally referred to as Pakhto). During the last decade terrorist activity and natural disasters has led to non-hygienic health caring facilities and low literacy rate (especially in females).

### Study Design

A survey was conducted between 15^th ^May and 31 July, 2009; in which different medical camps for IDPs were visited and primary data were collected. From all the patients who were attended at various medical camps by the medical staff, 950 volunteer patients were randomly selected for the study irrespective of their age, gender and location. Out of 950 volunteers, 320 from Swat, 200 from Dir (lower), 170 from Buner, 190 from Malakand and 70 were from Shangla district of Malakand Division. A predesigned questionnaire was filled out by all the volunteers, carrying information of age, gender, socioeconomic status, and various risk factors for the study purposes.

In the next phase of the study, ELISA and PCR analysis were performed to confirm the HBV infection status in the patients.

### Ethical approval

Signed consent of all the individuals involved in the study was received before enrollment, while the consent of the parents was recorded for all volunteers under 18.

### Blood samples collection

A 3 ml blood sample was collected in a vacutainer from each patient involved in the study. The serum was separated and stored at -20°C until further use for biochemical parameters, ELISA and PCR. Standard procedures for reducing contamination were strictly followed.

### Biochemical analysis

The liver function tests (LFTs) performed included alanine aminotransferase (ALT), alkaline phosphatase, aspartate aminotransferase (AST), and bilirubin using their relevant kits in a Shimadzu UV-Visible double beam Spectrophotometer1700 Pharma (Japan) as described in manufacture's manual.

### ELISAs for HBV

All the patients used in the liver function test were subjected to screening for HBsAg, HBeAg, anti-HBs and antiHBe antibodies using 3rd generation enzyme-linked Immunosorbant Assay (ELISA) (DRG Instruments, Germany) kits using the methodology described in the manufacturer's manual.

### HBV Qualitative PCR

In order to confirm infection of HBV, qualitative real PCR was performed, using Smart Cycler II Real-time PCR (Cepheid, USA) with HBV DNA qualitative kits (Sacace Biotechnologies, Italy) according to the kit protocol. A viral load of ≤ 2.0 m copies/mL was considered as 'cut off titre'.

### Data Analysis

The statistical analysis was performed using SPSS software.

## Results

Blood specimens of 950 HBV suspected patients were collected from the surveyed population of IDPs. At the time of specimen collection, demographic and socioeconomic status of each patient was recorded (Table 1). All the samples were screened with commercial ELISA kits for HBV; 52 (5.47%) samples were HBsAg/HBeAg positive, 235 (24.74%) Anti-HBS/Anti-HBe positive, while 309 (32.53%) and 37 (3.89%) blood samples, respectively, were positive for anti-HBs and HBsAg only.

**Table 1:** Demographic and socioeconomic status of the patients

District	*No.	Variables		Literacy		Socioeconomic status	
		
		♂	♀	^**1**^**L**	^**2**^**IL**	^**4**^**ML**	^**5**^**L**
**SWAT**	320	200	120	176	144	97	223

**Dir (Lower)**	200	150	50	60	140	60	140

**Buner**	170	90	80	65	105	30	140

**Malakand**	190	80	110	75	115	77	113

**Shangla**	70	30	40	20	50	20	50

**Demographic and socioeconomic status of the confirmed HBV**^**+ **^**patients**							

**Gender**		**Literacy**			**Socioeconomic status**		

♂	♀	Literate		Illiterate		Middle lower	Lower

157	43	69		131		57	143

*Number of Patients, ^1^Literate. ^2^Illiterate, ^4^Middle lower, ^5^Lower

### District wise Prevalence of HBV infection

**Figure 1:**
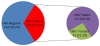
**HBV positive patients demographic characteristics**.

**Figure 2:**
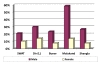
**Observed prevalence and gender wise distribution of HBV in the cohort of IDPs from different districts of Malakand division**.

A total of 200 (21.05%) samples gave positive PCR results for HBV infection (Figure 1). High incidence of HBV infection were reported in the cohort of IDPs from district Malakand (31.10%) and lower Dir (26%) as compared to district swat (18%), Buner (15.90%) and Shangla (15.71% ) (Figure 2).

### Gender disparity

The gender-wise incidence among the HBV infected persons showed that males were significantly more affected than the females (Figure 1 &2). In our studied population the PCR positive HBV samples included 157 (78.5%) males and 43 (21.5%) females (Figure 1). After statistical analysis male to female ratio was found to be 3.65:1.

### Age-wise prevalence

To study the age-wise prevalence, all the PCR positive samples were categorized into five age groups (Table 2). Interestingly it was observed that all the age groups were affected and a gradual rise in the prevalence was observed with an increase in age. The highest incidence rate of 29.13% was observed in the age group of 46-60 years while a lower incidence of 11.97% was observed in the age group of ≤ 15 years (Table 2).

**Table 2:** Prevalence of Hepatitis B virus among the various age groups

Age Groups	**Total obs**.	HBV patients cases	% Prevalence
**<15**	167	20	11.97%

**16-30**	150	23	15.33%

**31-45**	289	67	23.18%

**46-60**	254	74	29.13%

**>60**	90	15	16.66%

### Socioeconomic effects on the prevalence of HBV infection

The observed literacy rate was 34% and most of the patients (71.5%) were from a lower socioeconomic status (table 1). HBV positive subjects of the studied area belonged mostly to the rural areas. Most importantly, basic health facilities such as a basic health unit (BHU) or hospitals are far away from their reach or are of low hygienic standards. While in some areas good quality private hospitals are available but common people cannot afford these.

### Risk factors associated with HBV infection

Among the overall HBV positive cases, trends of sharing personal things i.e. shaving razor, ear and nose piercing needles, took brash and sewak, were very common. The study revealed that household contacts were the most frequent (51.5%) risk factors among the patients (Table 3). In this study the predisposing factors of hepatitis B included previous risks of dental procedures (41%), barber risk (32.5%), general surgery (15%), skin tattooing (11%) and sexual contact with hepatitis B positive partner (3.5%), as shown in table 3.

**Table 3:** Risk factors associated with Hepatitis B virus transmission

Risk Factors	Positive cases (*n = 200)		Total	Observed %
			
	Male	Female		
**Dental risk**	39	43	82	41%

**Barber risk**	65	-	65	32%

**G. surgery**	11	19	30	15%

**Household contact**	38	65	103	51.5%

**Skin tattooing**	3	19	22	11%

**Sexual contact**	3	4	7	3.5%

Sharing personal things**	61	84	145	72.5%

More than one category per subject is possible.

*Total number of HBV positive samples

** Shaving razor, ear and nose piercing needles, took brash and sewak

## Discussion

The purpose of this study was to investigate the prevalence of HBV in IDPs in the Malakand Division. This is one of the most backward and historically important rural areas of northern Pakistan. Most of the earlier studies conducted up to date in Pakistan have been focused on urban areas only. It has been confirmed by earlier studies that most HBV positive subjects belonged to the rural areas of low economic status [14]. More than 67.5% of the Pakistani population lives in rural areas [15]. It has been well documented that HBV infection is more prevalent in these low socioeconomic settings [14]. Because of this, a few years ago the Government of Pakistan launched a very effective immunization program against HBV to eliminate this silent killer from the country. Unfortunately that was limited to only urban areas, which is one of the main reasons, that the incidence of HBV is increasing in these localities. The present study might be helpful to draw the attention of local and international organizations towards these areas. The Malakand Division was taken as model because all its districts have very low literacy rate, are far-off from basic health facilities and are affected by tiresome and natural disasters (e.g. earthquake, floods).

The present study has confirmed about 21.05% prevalence of HBV in the surveyed population of IDPs. High prevalence of HBV was observed in IDPs from district Malakand and Dir (L) as compared to district Swat, Buner and Shangla. There were approximately three times as many males as females with HBV infection. Regarding gender-wise prevalence the frequency distribution of hepatitis B infection was found as higher in males (78.5%) as compared to the females (21.5%). It appears that male gender is playing an important role in the acquisition of HBV infection and might be caused by the higher exposure to the outside environment as compared to females who most of the days live at their houses. Barber shaving, homosexuality (male to male contact), and drug use are very common in these areas which strengthens the arguments for such high incidence of HBV among males. Similar results with 78.04% males and 21.95% of females HBV infections were observed by Shazi and Abbas during the comparison of risk factors associated with hepatitis B and C infection in patients at a liver stomach clinic Karachi [16]. High prevalence results of HBV in males compared to females have been observed in earlier studies in Pakistan [15,17]. Similar results have also been obtained in Bangladesh where the researchers reported high prevalence in males (67.86%) than females (32.14%) [18]. The aforementioned prevalence in men reflect the increased frequency of high risk behavior which might include multiple sexual partners, drug use and barber shaving as compared to women.

In almost all the age groups found to be affected, the prevalence rises gradually with age. High risk of infection was found in the older subjects as compared to the younger ones. The higher prevalence among older age groups may be attributed to the more frequent exposure to risk factors and prolonged HBV infection. This study has identified many of the significant risk factors for HBV infection. In this study, barber risk presented a major route of transmission because this exposure frequency was markedly higher (32%) in male individuals who routinely shaved with community barbers. Janjua and Nizamy (2004) reported that 46% of the barbers in Pakistan reuse blades, concluding that the possibility of transmission increases with the frequency of reused blades [19]. According to one study conducted by Usman and his colleagues in 2003, the most frequent risk factor contributing to HBV infection is barber risk and it accounts for 47.6% of the infection [17]. The statistics of their results deviate from our study because the trend has changed substantially during the last few years and barbers have become more aware about HBV infection and have started using disposable blades and razors.

The contaminated dental instruments also play an important role in HBV infection because of the presence of HBsAg in the saliva of acute and chronic hepatitis B patients [20]. According to our findings, patient with prior history of dental treatment were mostly in their old ages (~41%). Most of the patients were from low economics status and were attended by local medical practitioners. In fact these practitioners mostly do not possess equipment for proper autoclaving, and some are illiterate and do not know what autoclaving is. Thus the lack of proper sterilization techniques and reuse of contaminated dental equipment might be a reason for such high rate of prevalence in these areas. These results are also supported by shazi and Abass (2006) who observed similar results (43.9%) for the dental risks [16]. Usman *et al *(2003) reported a high percentage (60%) of HBV patient with prior history of dental surgery as compared to current study [17]. Another predisposing risk factor of hepatitis B infection with previous history of surgery was positive in 15% of the patients. Similar results of 21.9% and 21.43% were reported by Shazi and Abass (2006) and Khan *et al *(2007) [16,21].

Sharing personal items (72.5%) such as tooth brushes, sewak (miswak), and shaving razors and frequent household contact (51.5%) have also been identified as major risk factors for HBV infection. Tanveer and colleagues in 2008 reported that there is a high habit (37.5%) of sharing personal belongings in the infected persons that could account for the high risk of infection [22]. It is likely that the high prevalence reported in household contacts in Pakistan may be due to the fact that they would be exposed to the same community risk factors as the index patients, rather than intra-household transmission per se. In contrast, HBV intra-familial transmission is well documented outside of Pakistan, and HBV is far more infectious as compared to HCV or HIV [23-25]. Skin tattooing and sexual contact with hepatitis positive partner contribute 11% and 3.5% in the spread of HBV infection respectively.

## Conclusions

The study revealed the seroprevalence of HBV among the various age groups along with the risk factors. High prevalence was observed in males as compared to females. The infection was more common in the older groups. We have also identified the major risk factors in this study which include barber shave, dental equipment & procedures, sharing personal items (e.g. tooth brash, sewak) and household contacts. Timely identification of people suffering from HBV infection is mandatory to control its rapid spread. Stringent aseptic measures should be adopted during surgical and dental procedure. More emphasis should be given to the preventive measures which include screened blood transfusion, safe sexual practice, use of disposable syringes and razors and proper disposal of contaminated materials. Importantly the Government authorities and non government organizations (NGOs) should take aggressive steps to educate and create awareness among the local population about the possible risk factors to avoid the transmission of HBV infection and extend the benefits of immunization to rural areas.

## List of abbreviations

HBV: Hepatitis B virus; IDPs: internally displaced persons; HCC: hepatocellular carcinoma; NGOs: non government organizations; HBsAg: Hepatitis B surface antigen; LFTs: liver function tests; ALT: alanine aminotransferase; AST: aminotransferase; ELISA: enzyme-linked Immunosorbant Assay.

## Competing interests

The authors declare that they have no competing interests.

## Authors' contributions

FK, HA and HK performed lab work. HA and KS prepared and organized the final version of the manuscript. MI and MAK were the principal investigators and provide support and facilities to complete this work. All authors approved the manuscript.

## Authors' information

Fawad Khan (M.Phil Molecular Biology), Haji Akbar (M. Phil Molecular Biology), Hayat Khan (M.Phil Molecular Biology) and Khuram Shehzad (MS Bioinformatics) are PhD scholars. Mahmood Akhtar Kayani (PhD Cancer Genetics) is HoD department of Biosciences, COMSATS, institute of IT, Islamabad, while Muhammad Idrees (PhD Molecular Biology) is Principal Investigator at CEMB, University of the Punjab, Lahore

## Pre-publication history

The pre-publication history for this paper can be accessed here:

http://www.biomedcentral.com/1471-2334/11/176/prepub
